# *Novel Seed Size*: A Novel Seed-Developing Gene in *Glycine max*

**DOI:** 10.3390/ijms24044189

**Published:** 2023-02-20

**Authors:** Mingxia Zhang, Rui Dong, Penghui Huang, Mingyang Lu, Xianzhong Feng, Yongfu Fu, Xiaomei Zhang

**Affiliations:** 1 Moa Key Lab of Soybean Biology (Beijing), National Key Facility of Crop Gene Resource and Genetic Improvement, Institute of Crop Sciences, Chinese Academy of Agricultural Sciences, Beijing 100081, China; 2 Research Center for Intelligent Computing Platforms, Zhejiang Laboratory, Hangzhou 311121, China; 3 The Key Laboratory of Plant Resources Conservation and Germplasm Innovation in Mountainous Region (Ministry of Education), Institute of Agro-Bioengineering, Guizhou University, Guiyang 550025, China

**Keywords:** seed size, *Novel Seed Size* (*NSS*), seed coat, flavonoids, T-DNA mutant

## Abstract

Soybean-seed development is controlled in multiple ways, as in many known regulating genes. Here, we identify a novel gene, *Novel Seed Size* (*NSS*), involved in seed development, by analyzing a T-DNA mutant (*S006*). The *S006* mutant is a random mutant of the *GmFTL4pro:GUS* transgenic line, with phenotypes with small and brown seed coats. An analysis of the metabolomics and transcriptome combined with RT-qPCR in the *S006* seeds revealed that the brown coat may result from the increased expression of *chalcone synthase 7/8* genes, while the down-regulated expression of *NSS* leads to small seed size. The seed phenotypes and a microscopic observation of the seed-coat integument cells in a CRISPR/Cas9-edited mutant *nss1* confirmed that the *NSS* gene conferred small phenotypes of the *S006* seeds. As mentioned in an annotation on the Phytozome website, *NSS* encodes a potential DNA helicase RuvA subunit, and no such genes were previously reported to be involved in seed development. Therefore, we identify a novel gene in a new pathway controlling seed development in soybeans.

## 1. Introduction 

Soybean (*Glycine max* (L.) Merr.) is one of the most important multiuse crops, providing plant proteins and edible oils for humans and animals [1]. With the continuous growth in the population, the global demand for soybean is also increasing rapidly. Thus, the improvement of soybean yield is urgently needed. The yield-component factors of soybean include plant architecture, the number of seeds per pod, and seed weight/size [2]. Seed size not only determines yield but also influences plant fitness and adaption [3]. Therefore, the identification of genes controlling seed size and the exploration of the relevant mechanism are among the focuses of soybean research. 

Until now, several genes controlling seed size have been isolated and characterized in soybean [4]. The *BIG SEEDS1* (*BS1*) gene, encoding a plant-specific transcription regulator, has been reported to control seed size and weight through a regulatory module that targets primary cell proliferation in soybean [5]. In addition, *GmKIX8-1*, a negative regulator for cell proliferation, is also involved in the control of seed size [6]. Cytochrome-P450 family members have been confirmed to regulate organ size and development [7]. Furthermore, *GmCYP78A10*, *GmCYP78A57*, *GmCYP78A70*, and *GmCYP78A72* all demonstrate positive control over seed size in soybean [8,9]. A putative soybean invertase inhibitor, *GmCIF1*, participates in controlling seed maturation through specifically depressing CWI (cell-wall invertase) activities [10]. A novel PSK-encoding gene, *GmPSKγ1*, increases seed size by encouraging cell expansion [11]. A SPINDLY-like gene, *GmSSS1*, positively regulates soybean-seed size by influencing cell expansion and cell division [12]. In addition, some regulatory genes with natural allelic variations, which relate to seed size and quality, were reported recently [4]. A *phosphatase 2C-1* (*PP2C-1*) allele contributes to increasing seed weight/size by enhancing integument-cell size and activating seed-trait-related genes [13]. In addition, *GmSWEET10a* and *GmSWEET10b*, which contribute to sugar allocation from seed coat to embryo, determine the oil and protein contents and seed size in soybean [14,15,16]. Furthermore, *GmGA3ox1*, which encodes gibberellin 3β-hydroxylase, positively regulates soybean-seed weight [17]. A semi-dominant locus, *ST1* (*Seed Thickness 1*), affects seed thickness and oil content [18], and *GmPDAT* is responsible for the seed size and oil content in soybean [19]. The discovery of more genes related to seed size will accelerate soybean breeding for yield improvement [4].

Here, we identified a T-DNA insertion mutant, *S006*, which has brown seed coats and small seeds. Metabolomes revealed that flavonoids accumulated in the *S006* seeds, and a transcriptome analysis showed that the up-regulated expression of *CHS7/8* might result in the pigmentation of seed coats in the *S006* mutant. The resequencing data showed that T-DNA is inserted into chromosome 8 of the soybean genome, which affects the expression levels of flanking genes, including a down-regulated gene, *Glyma.08G309000*. The CRISPR/Cas9-edited mutants of the *Glyma.08G309000* gene demonstrated similar phenotypes of small seeds to the *S006* mutant seeds. Therefore, *Glyma.08G309000* was named as *Novel Seed Size* (*NSS*). Altogether, we identified a novel gene controlling seed development in soybean.

## 2. Results

### 2.1. Phenotypes of S006 Mutant

**Figure 1:**
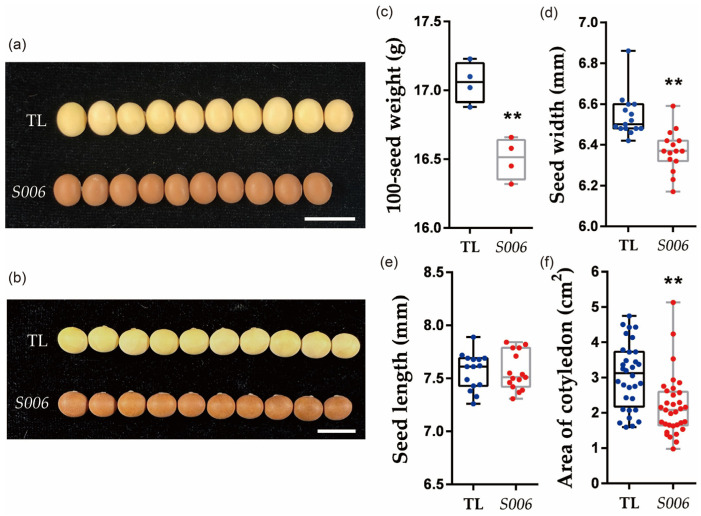
Comparative analysis of phenotypes between *S006* mutant and wild-type seeds. (**a**) Image of seeds showing decreases in width in *S006* mutant (10 seeds are shown in each case); (**b**) image of seeds showing no significant changes in length in *S006* mutant; (**c**) 100-seed weight was significantly decreased in *S006* mutant; (**d**) seed width significantly decreased in *S006* mutant.; (**e**) there was no significant difference in seed length between *S006* mutant and wild type; (**f**) cotyledon area significantly decreased in *S006* mutant. Asterisks indicate a significant difference according to Student’s *t*-test (**, *p* < 0.01).

In previous studies, we created a binary expression vector, which carried a *GmFTL4* (*Glyma.16G044100*) promoter driving the *GUS* reporter gene to elucidate the expression specificity of *GmFTL4* (Figure S1), and obtained five independent stable transgenic soybean lines by *Agrobacterium*-mediated transformation. Unexpectedly, we found a unique line with brown seed coats and small seeds (named *S006*), which was different from the other transformed lines, which had normal yellow seed coats. Compared with wild-type plants (TL), seeds of the *S006* mutant exhibited significantly lower seed width (Figure 1a,d), which resulted in a decrease in 100-seed weight in this mutant (Figure 1c). Consistent with these results, the cotyledon area of the *S006* also decreased compared with the wild-type plants (Figure 1f). However, the plant height, number of pods per plant and number of seeds per plant showed no significant difference between the *S006* mutant and the wild type (Figure S2).

**Figure 2:**
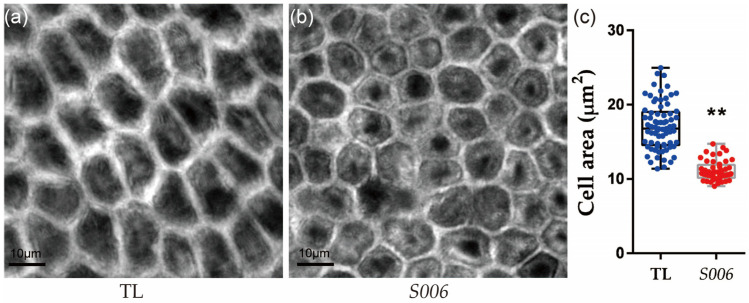
Comparison of seed-integument cells between *S006* and wild-type plants. (**a**) Image of integument cells in wild type; (**b**) image of integument cells in *S006* mutant; (**c**) statistical analysis of cell area of *S006* and wild-type seeds. Asterisks indicate a significant difference according to Student’s *t*-test (**, *p* < 0.01).

Because the seed size was greatly affected by the integument size, which is determined by cell proliferation and cell expansion [13,20], the areas of the outer-integument cells in the seeds of the wild-type and *S006* plants were compared. We found that the area of the outer-integument cells in the seeds of the *S006* mutant was significantly decreased compared with that of the wild type (Figure 2a–c). 

### 2.2. Metabolite Profiling of S006 Mutant and Wild-Type Seeds

**Figure 3:**
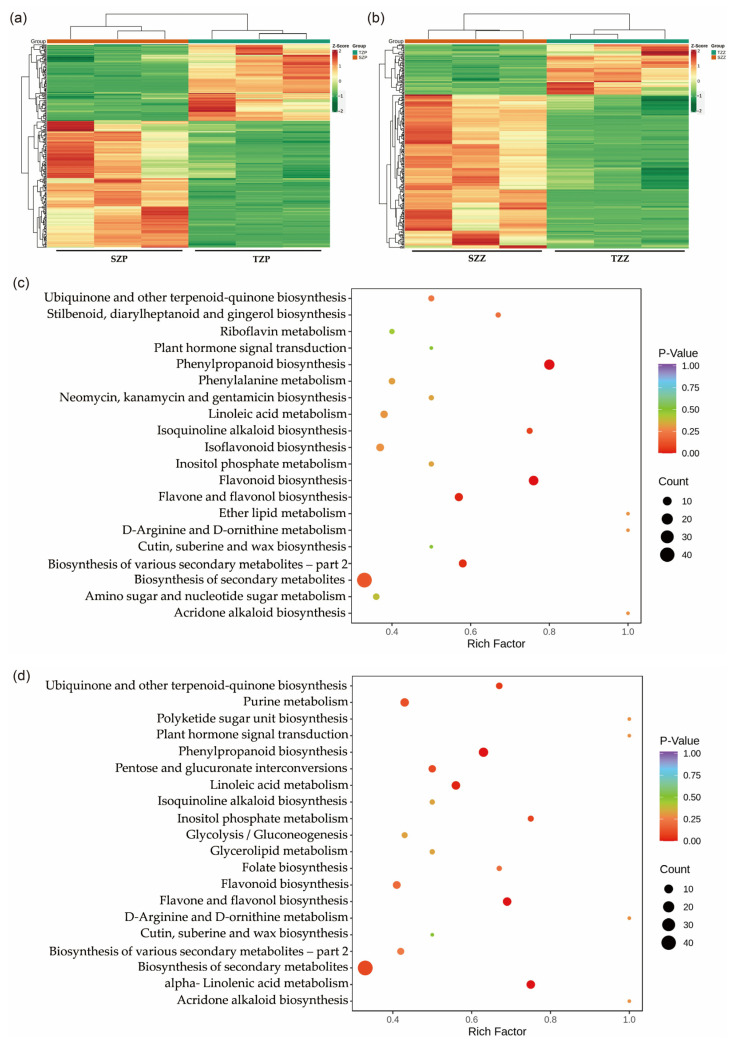
Differentially accumulated metabolites among seed coats and cotyledons of *S006* vs. TL. (**a**) A heat map of differential metabolites among SZP vs. TZP; (**b**) a heat map of differential metabolites among SZZ vs. TZZ; (**c**) KEGG-pathway-enrichment analysis of differential metabolites among SZP vs. TZP; (**d**) KEGG-pathway-enrichment analysis of differential metabolites among SZZ vs. TZZ.

To further explore the changes in metabolism in the *S006* mutant seeds, differential metabolites were analyzed among the seed coats of the *S006* (SZP) versus the TL (TZP) and the cotyledons of the *S006* (SZZ) versus the TL (TZZ). In total, 255 significant diverse metabolites were identified among the SZP versus the TZP, comprising 159 up-regulated and 96 down-regulated metabolites (Figure S3a). Most of these up-regulated metabolites were flavonoids, while the down-regulated metabolites were phenolic acids (Table S1). Furthermore, a total of 268 significant diverse metabolites were identified among the SZZ versus the TZZ, with 202 up-regulated and 66 down-regulated metabolites (Figure S3b). Compared with the SZP versus the TZP, the up-regulated metabolites of the SZZ versus the TZZ included lipids, organic acids, amino acids and derivatives, in addition to flavonoids (Table S2). The heat maps of the differential metabolites among these two combinations also clearly indicated the similar variations (Figure 3a,b). 

To further investigate the biological processes involved, the differential metabolites were assigned to KEGG pathways. As shown in Figure 3c, the differential metabolites of the SZP versus the TZP were significantly involved in flavonoid, flavone, flavonol, and phenylpropanoid biosynthesis. In addition, the differential metabolites of the SZZ versus the TZZ were involved in the biosynthesis of ubiquinone and other terpenoid quinones, as well as linoleic acid and alpha-linolenic-acid metabolism (Figure 3d). Seed-coat color is affected by many factors, among which the most important is the flavonoids [21,22]. Based on the above results, we speculated that the pigmentation of the *S006* seed coat was caused by the accumulation of flavonoids. 

### 2.3. Up-Regulated Expressions of CHS-Related Genes Induced the Pigmentation of Seed Coat in S006 Mutant 

**Figure 4:**
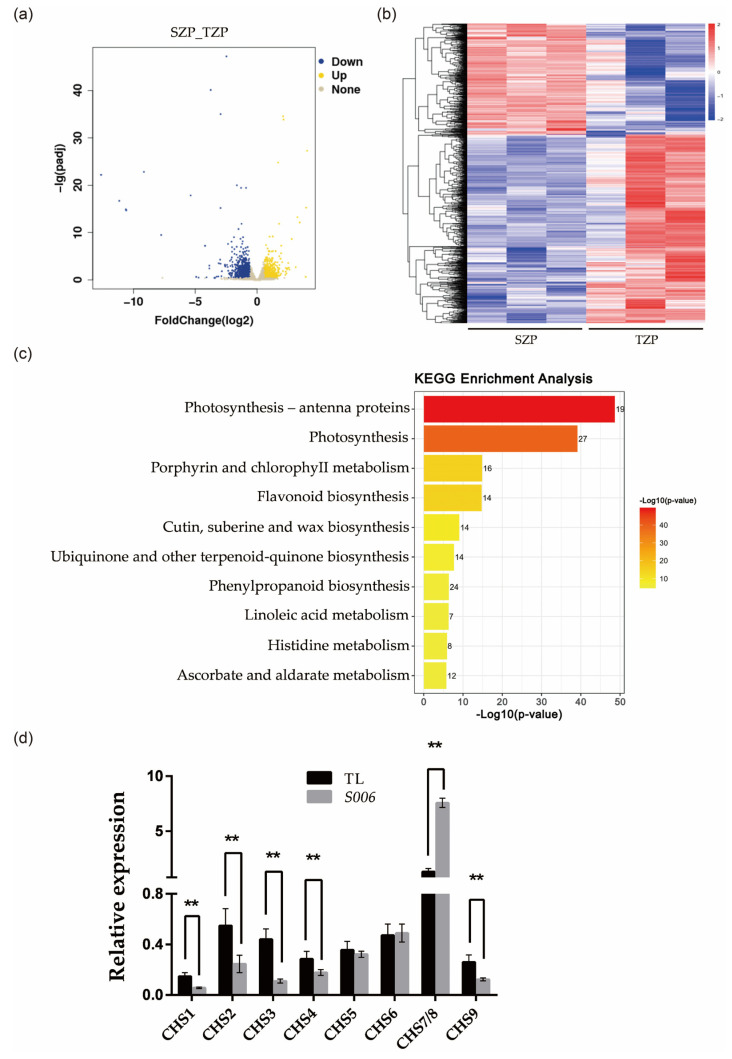
Transcriptome analysis of *S006* and wild type. (**a**) A volcano plot of DEGs between *S006* and wild type; (**b**) a heat map of DEGs between *S006* and wild type; (**c**) the ten KEGG pathways that were the most enriched in DEGs; (**d**) the RT-qPCR verified the expression of nine *CHS* genes. Asterisks indicate a significant difference according to Student’s *t*-test (**, *p* < 0.01).

To elucidate the mechanisms underlying the changes in color and cell area of the seed coat in the *S006* mutant, the transcriptomes of the seed coat of the wild type and the *S006* mutant were compared by RNA-seq analysis. In particular, the expressions of 2225 genes (foldchange ≥ 1.5, *p* < 0.05, Table S3) were significantly changed in the *S006* mutant, including 824 up-regulated genes and 1401 down-regulated genes (Figure 4a,b). This group of significantly changed genes contains a cluster of bHLH transcription factors (Figure S4), indicating that the phenotypic changes in the *S006* mutant may be correlated with the bHLH transcription factors. To further assess the biological functions of the differentially expressed genes (DEGs), we performed gene ontology (GO) and top-10-KEGG analyses. The results showed that the DEGs in the *S006* mutant were mainly enriched in three pathways: photosynthesis, porphyrin and chlorophyll metabolism, and flavonoid biosynthesis (Figure 4c). Next, we selected some genes related to flavonoid biosynthesis for the RT-qPCR analysis to confirm the RNA-seq data. These genes included *CHS1* (*Glyma*.*08G109400*), *CHS2* (*Glyma*.*05G153200*), *CHS3* (*Glyma*.*08G109300*), *CHS4* (*Glyma*.*08G110700*), *CHS5* (*Glyma*.*08G109200*), *CHS6* (*Glyma*.*09G075200*), *CHS7/8* (*Glyma*.*01G228700* and *Glyma*.*11G011500*), and *CHS9* (*Glyma*.*08G109500*). The RT-qPCR results were in agreement with the RNA-seq data (Figure 4d). Combined with previous research results [23,24], we assumed that the up-regulated transcription of *CHS7/8* resulted in the accumulation of flavonoids in the seed coat, thus pigmenting the seed coat in the *S006* mutant.

### 2.4. T-DNA Insertion in Genome Caused the Expression Changes in Flanking Genes

**Figure 5:**
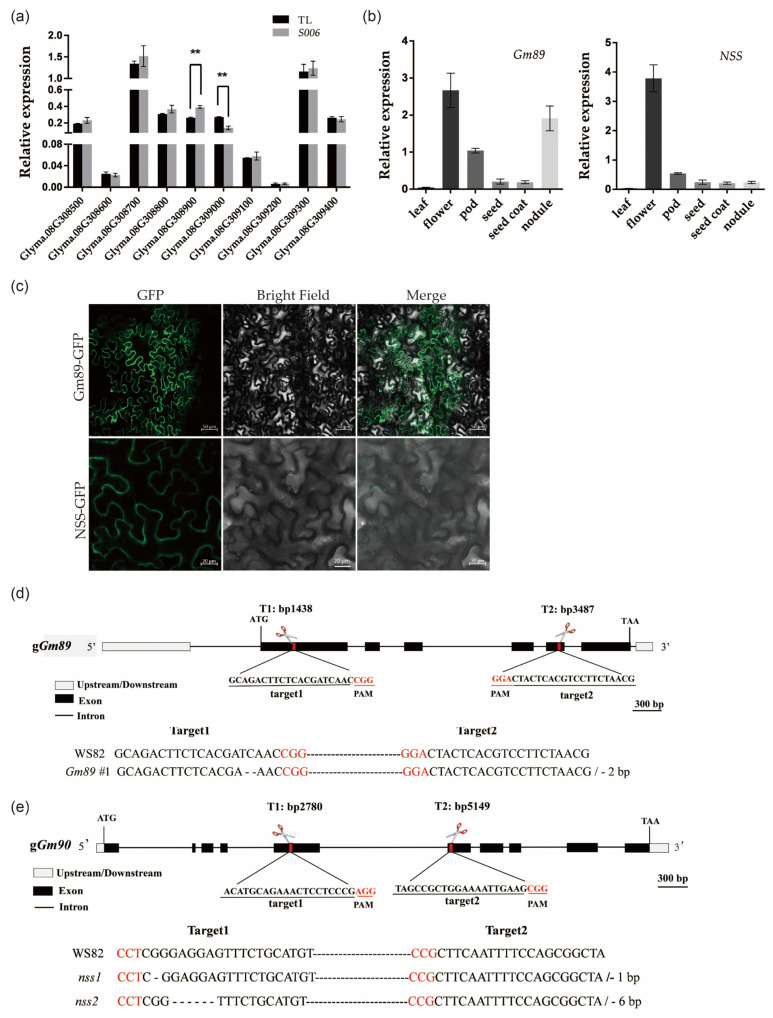
Expression patterns, protein subcellular localization, and mutant constructions of *Gm89* and *NSS* genes. (**a**) Flanking-gene expressions of T-DNA insertion site in *S006* mutant. Asterisks indicate a significant difference according to Student’s *t*-test (**, *p* < 0.01); (**b**) expression patterns of *Gm89* and *NSS*. (**c**) subcellular localization of Gm89 and NSS proteins. Bars for upper row = 50 μm, bars for lower row = 20 μm; (**d**) identification of *Gm89* mutant. The red letters represent the PAM site; (**e**) identification of *nss* mutants. The red letters represent the PAM site.

Because the phenotypes of the *S006* mutant were different from those of other transformed lines, we speculated that its phenotype variation may be related to the insertion of T-DNA. To investigate the molecular mechanism for these variations, the T-DNA insertion site was identified firstly by resequencing. The results showed that T-DNA inserted in 42,785,587 bp of chromosome 8 in soybean genome (Figure S5). Next, the expression levels of 10 genes around upstream and downstream of this insertion site were analyzed. The results of the RT-qPCR showed that the *Glyma.08G308900* (*Gm89*) was up-regulated and the *Glyma.08G309000* (*Gm90*) was down-regulated (Figure 5a). Therefore, these two genes were employed as candidate genes for further study. As shown by the data below, *Gm90* may be related to seed development; thus, it was renamed as *Novel Seed Size* (*NSS*).

### 2.5. Expression Patterns, Protein Subcellular Localizations and Mutant Constructions of Gm89 and NSS

To study the specific expression profiles of the *Gm89* and *NSS* genes in different organs, we examined the transcript accumulation in the soybean organs by RT-qPCR. The results showed that the flowers accumulated the highest amount of *Gm89* and *NSS* transcripts. Furthermore, the nodules had high levels of *Gm89* transcripts. In addition, *Gm89* and *NSS* transcripts were also detected in the pods, seeds, and seed coats (Figure 5b). The data implied wide roles of *Gm89* and *NSS* in soybean development.

To visualize the subcellular localization of the Gm89 and NSS proteins, fusion genes of *Gm89:GFP* and *NSS:GFP* driven by a *35S* promoter were constructed individually and then infiltrated into *Nicotiana benthamiana* leaves mediated by *Agrobacterium tumefaciens*. The GFP fluorescence signals in the cytoplasm (Figure 5c) indicated that two genes may function in the cytoplasm.

Next, we constructed mutants of *Gm89* and *NSS* in the WS82 background by normal CRISPR/Cas9 technology to unveil their respective biological functions. The PCR amplifications were performed to screen mutant lines of the *Gm89* and *NSS*. In particular, *Gm89#1* lost 2 bp at target 1, leading to the early termination of the translation (Figure 5d). We also obtained two *nss* mutants. The *nss1* mutant was terminated early due to the loss of 1 bp at target 1, whereas *nss2* only missed two amino acids at target 1 (Figure 5e). Next, the *Gm89#1*, *nss1*, and *nss2* mutants were used for a phenotypic analysis.

### 2.6. NSS Is Involved in Regulating Seed Size in Soybean

**Figure 6:**
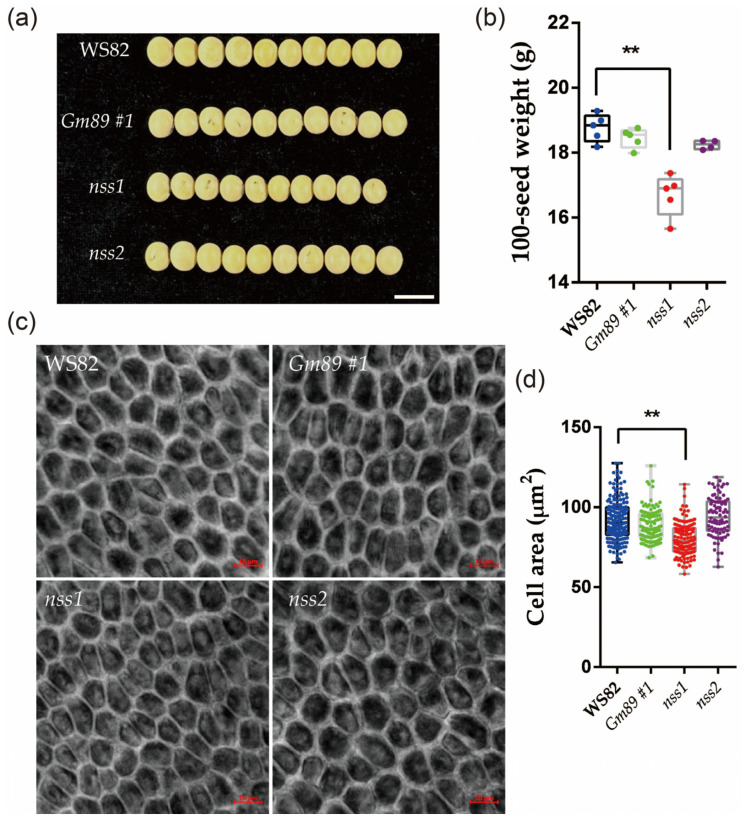
Comparative analysis of phenotypes between mutants and wild-type seeds. (**a**) The seed size decreased in width in *nss1* mutant (10 seeds are shown in each line); (**b**) the 100-seed weight was significantly decreased in the *nss1* mutant; (**c**) the integument cells in mutants and wild-type seeds. Bars = 10 μm; (**d**) the cell area of mutants and wild-type seeds. Asterisks indicate a significant difference according to Student’s *t*-test (**, *p* < 0.01).

In order to further clarify the functions of the *Gm89* and *NSS* genes in the *S006* phenotypes, the agronomic traits of the transformed plants were investigated. The results showed that the 100-seed weight of *nss1* was significantly lower than that of WS82, while the 100-seed weight of *Gm89#1* and *nss2* was not significantly different from that of the wild type (Figure 6a,b). The integument cells of the mutant seeds were further observed by confocal microscopy, and the cell area of *nss1* was significantly reduced compared with the wild type, indicating that *NSS*-gene mutation contributed to *S006*-seed-size phenotypes. The cell area of *Gm89#1* and *nss2* was not significantly different from that of WS82, which was consistent with the phenotypes of seed size, suggesting that neither the *Gm89* gene nor the two-amino-acid deletion in *nss2* produced seed-size phenotypes. 

Because the seeds of the *nss1* mutant showed small sizes, lower 100-seed weights and reduced areas of integument cells (Figure 6), it is suggested that the *NSS* gene is involved in controlling seed size. A sequence analysis provided by Phytozome showed that *NSS* encoded a functional unknown protein, which contained a peptidase-c1 domain as a potential DNA helicase RuvA subunit and may be involved in apoptosis. In previous studies [5,6,7,8,9,10,11,12,13,14,15,16,17,18,19], some genes encoding transcription regulators or enzymes were demonstrated to control seed size. However, no such genes or pathways have been reported to be related to seed development. From these results, we concluded that *NSS* is a novel gene involved in regulating seed size.

## 3. Discussion

Flavonoids are the most important components that affect seed-coat color. They mainly include anthocyanins, proanthocyanidins, flavonoids, isoflavones, and xanthones [25]. The metabonomic analysis of the *S006* mutant showed that more than 200 different metabolites were detected in the seed coat, among which flavonoids and phenolic acids accounted for the largest proportion. The KEGG pathway was significantly enriched in the synthesis of phenylpropanoids and flavonoids (Figure 3c), and the content of dihydrokaempferol was significantly increased, indicating that the anthocyanin formation was significantly increased in the *S006* mutant, resulting in the pigmentation of the seed coat. In addition, many flavonoids were also among the differential metabolites in the cotyledons, further indicating that the flavonoid metabolism in the seeds changed significantly in the *S006* mutant.

The endogenous RNA interference (RNAi) of *chalcone synthase* (*CHS*) genes inhibits pigmentation of seed coats in soybean [26]. It was found that *CHS* genes formed *CHS1*-*CHS3*-*CHS4* + 5.87 kb + *CHS1*-*CHS3*-*CHS4* inverted tandem repeat structure (GmIRCHS) on the chromosome [27]. The *CHS3* is a pseudogene due to partial truncation and forms an inverted repeat sequence (IR), which produces a dsRNA region and induces the post-transcriptional gene silencing of the *CHS* genes [27,28]. The pigmentation in seed coats is related to the release of *CHS7/8* genes from the silencing effect [24,29]. In addition, although *CHS* genes are expressed in both the seed coat and the cotyledon, the RNAi of *CHS* genes occurs specifically in the seed coat [26]. The results of our transcriptome analysis showed that the pigmentation of the *S006* mutant seed coat may have been due to the change in the expression levels of the genes related to the flavonoid-synthesis pathway (Figure 4c). Through the RT-qPCR experiments, it was verified that *CHS1*, *CHS2, CHS3*, *CHS4*, and *CHS9* were significantly down-regulated and that *CHS7/8* were significantly up-regulated in the *S006* mutant (Figure 4d). Consistent with previous research results, the up-regulated expression of *CHS7/8* may be the main reason for the pigmentation of the *S006* seed coat. However, not only the *nss* mutant but also the *Gm89#1* mutant appeared to have a normal seed-coat color, suggesting these two genes did not participate in the pigmentation of the *S006* mutant’s seed coat.

No DEGs of known genes related to cell division or expansion were found in the transcriptome analysis, despite the reduction in the outer-integument-cell area in the *S006* mutant. The expression levels of *Gm89* and *NSS* were confirmed to have changed in the *S006* mutant; however, only the *nss1*, and not the *Gm89* mutant seeds showed small size, lower 100-seed weight, and reduced area of integument cells (Figure 6), suggesting that the *NSS* gene is a novel gene controlling seed development. The *NSS* gene encodes a functional unknown protein, which contains a peptidase-c1 domain as a potential DNA helicase RuvA subunit and may be involved in apoptosis. There are no reports showing genes with similar functions participating in seed development [5,6,7,8,9,10,11,12,13,14,15,16,17,18,19]. Since the *nss1* mutant exhibited small integument cells, the *NSS* gene may be related to cell expansion. Regarding the encouragement of cell expansion by *GmPSKγ1* and *GmSSS1* [11,12], it would be of interest to investigate the relationship between them and their functions in cell expansion in seed coats.

## 4. Materials and Methods

### 4.1. Plant Materials and Growth Conditions

The *S006* homozygous mutants were generated via *Agrobacterium*-mediated transformation in the *G. max* (L.) Mer. cultivar Tianlong1 (TL), and TL wild-type plants were used as controls. The *Gm89#1*, *nss1*, and *nss2* genetically modified lines were generated in Williams 82 (WS82) background mediated by *Agrobacterium tumefaciens*, and WS82 used as control. All soybean plants were grown in a growth room under short-day conditions (8-hour-light/16-hour-dark cycle) at 25 ± 1 °C with light illumination. *Nicotiana benthamiana* plants were grown in a growth room at 22 ± 1 °C with a 16-hour-light/8-hour-dark cycle. The light conditions were from a LED light source (GreenPower LED top lighting, PhilipsHorticulture LED). 

### 4.2. Vector Construction and Soybean Transformation

The 8.9 kb from start coden ATG of *GmFTL4* promoter was amplified from soybean-cultivar TL1 genome and then inserted into the Fu79-*GUS* vector [30] to fuse with GUS reporter gene. The binary vector, Fu39-2:*GmFTL4_pro_:GUS*, was constructed through LR reaction (Invitrogen, Carlsbad, CA, USA) and transformed into TL1 following the cotyledonary node method [31]. 

The network-based tool CRISPR-P (http://crispr.hzau.edu.cn/cgi-bin/CRISPR2/CRISPR, accessed on 21 December 2020) was used to design sgRNAs for *Gm89* and *Gm90* (*NSS*) genes [32]. For soybean-genome editing, two entry vectors that individually expressed the Cas9 protein [33] and sgRNAs were constructed, as in our previous report [34]. Next, the genome-editing vectors were introduced into *Agrobacterium tumefaciens*. Soybean transformations were completed by Wuhan Edgene Bio-tech CO., LT (Edgene, Wuhan, Hubei, China).

### 4.3. RNA-Extraction and -Expression Analysis

Total RNA of seeds or other tissues were extracted using an EasyPure RNA Kit (TransGen Biotech, Beijing, China). About 1 μg of total RNA was used in reverse transcription (Tiangen, China) for SYBR detection of RT-qPCR products. The ChamQTM SYBR qPCR Master Mix (High ROX Premixed) (Vazyme, Nanjing, Jiangsu, China) was used to detect the expression levels of genes. In the experiment, *GmUKN2* (*Glyma.06G04180*) was used as the internal reference gene [35] for the detection of gene expressions. All primers are listed in Supplemental Table S4.

### 4.4. Subcellular Localization

The *Gm89:GFP* and *NSS:GFP* fusion genes driven by the *Cauliflower mosaic* virus 35S promoter were transiently expressed in the leaves of 2- to 4-week-old *N. benthamiana* leaves. The fluorescence signals were visualized by Zeiss LSM700 confocal laser scanning microscope.

### 4.5. Metabolite Detection and Data Analysis

Seed coats and cotyledons from mature seeds of *S006* and TL1 were collected and stored in refrigerator at −80 °C. We used MetWare (Wuhan, China) to perform metabolite identification and quantification according to their standard procedures. 

### 4.6. Transcriptome Analysis

In R5 stage, seed coats of *S006* and TL1 were collected and stored in refrigerator at −80 °C. Total RNA extraction, sequencing with an Illumina HiSeq instrument and data analysis were performed by Annoroad Gene Technology (Beijing, China).

## 5. Conclusions

We identified a novel gene participating in seed development from a random T-DNA mutant, whose mutation resulted in small integument cells, small seeds, and lower 100-seed weights. 

## Data Availability

Data are contained within the article and Supplementary Materials.

## Supplementary Materials

The following supporting information can be downloaded at: https://www.mdpi.com/article/10.3390/ijms24044189/s1**.**
