# Supplementary material for: Novel Seed Size: A Novel Seed-Developing Gene in Glycine max
Source: Int J Mol Sci. 2023 Feb 20;24(4):4189. doi: 10.3390/ijms24044189 (PMC9967547; doi:10.3390/ijms24044189)
Supplement: Supplementary file 1 [file ijms-24-04189-s001.zip › Supplementary data.pdf]

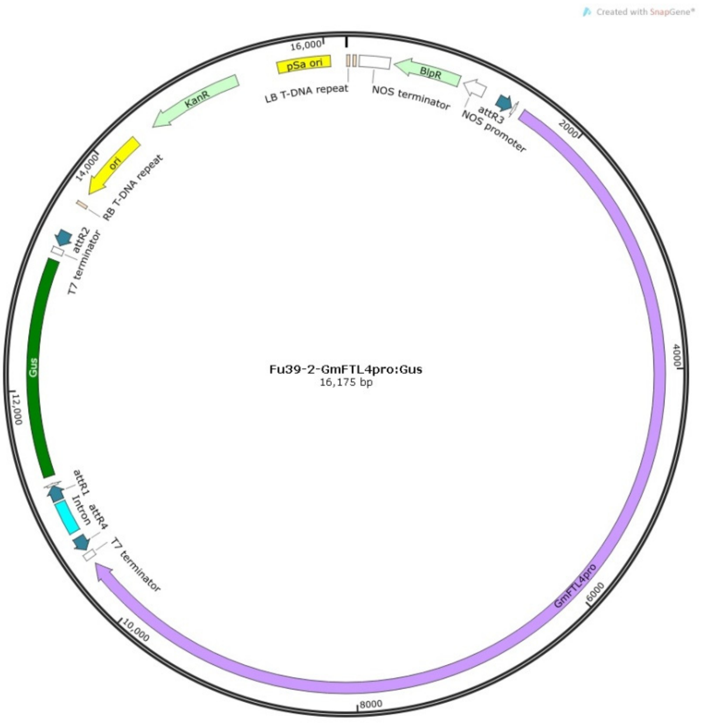

Figure S1. The vector map of Fu39-2-*GmFTL4pro*:*Gus*

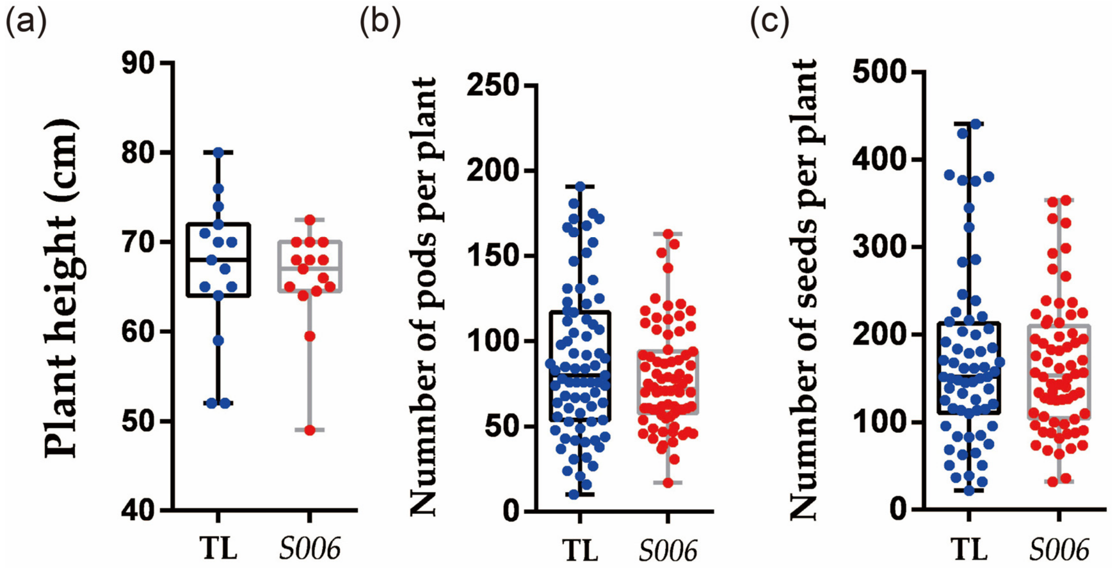

Figure S2. Comparative analysis of the plant height, number of pods per plant and number of seeds per plant between the *S006* mutant and WT. (a) No significant difference was shown in plant height between the *S006* mutant and wild type; (b) no significant difference was shown in number of pods per plant between the *S006* mutant and wild type; (c) no significant difference was shown in number of seeds per plant between the *S006* mutant and wild type.

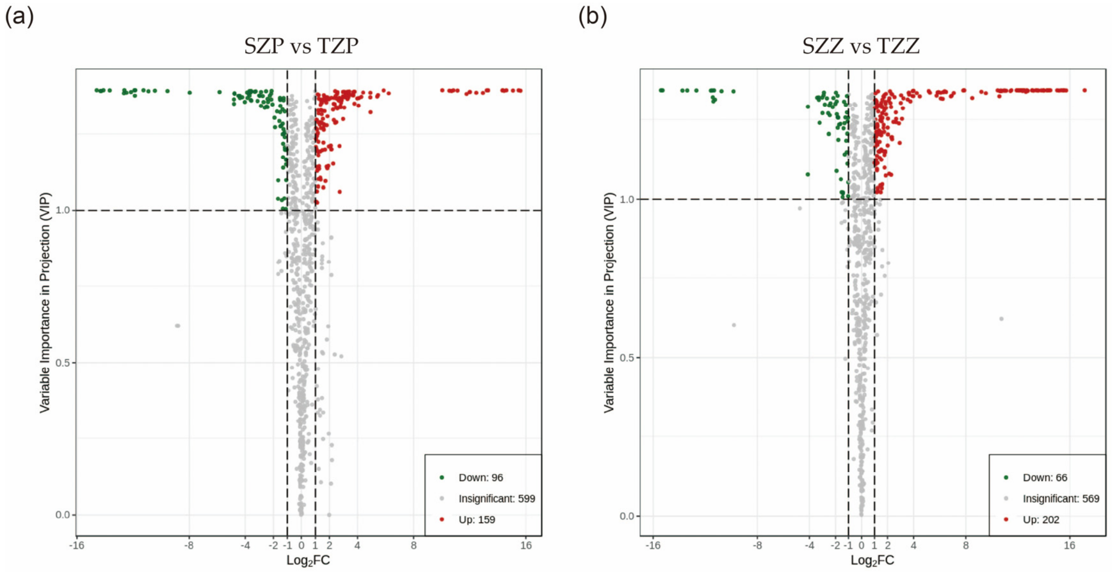

Figure S3. Volcano plots of differential metabolites of SZP vs. TZP and SZZ vs. TZZ. (a) A volcano plot of differential metabolites of SZP vs. TZP; (b) a volcano plot of differential metabolites of SZZ vs. TZZ.

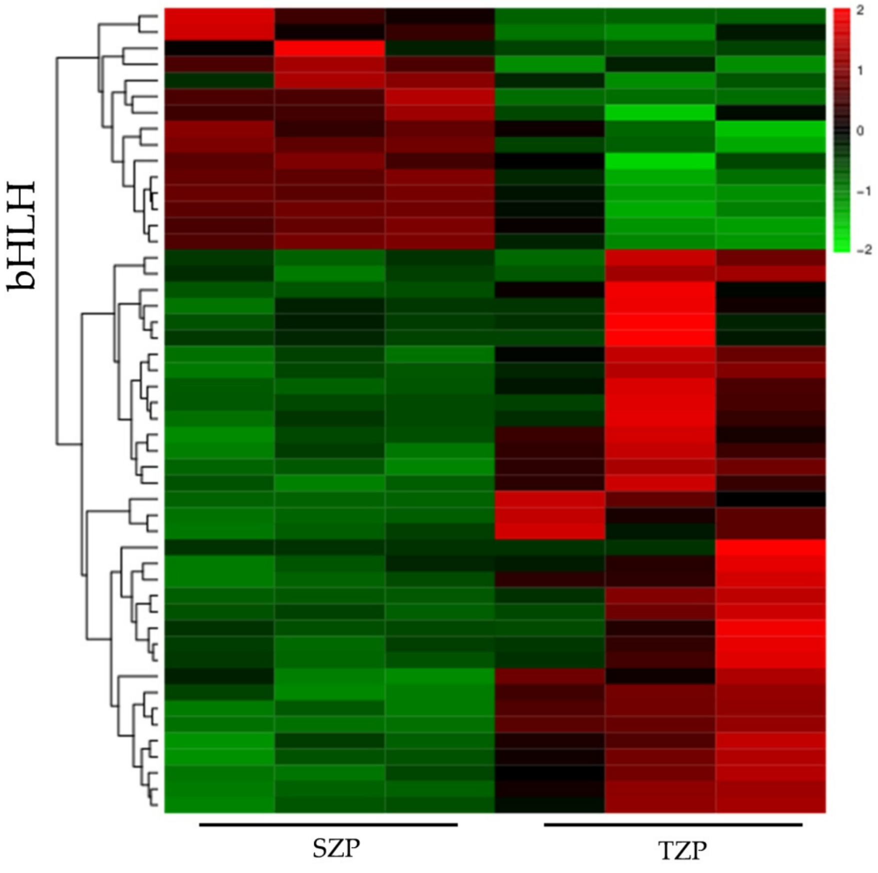

Figure S4. The bHLH transcriptional factors identified among DEGs.

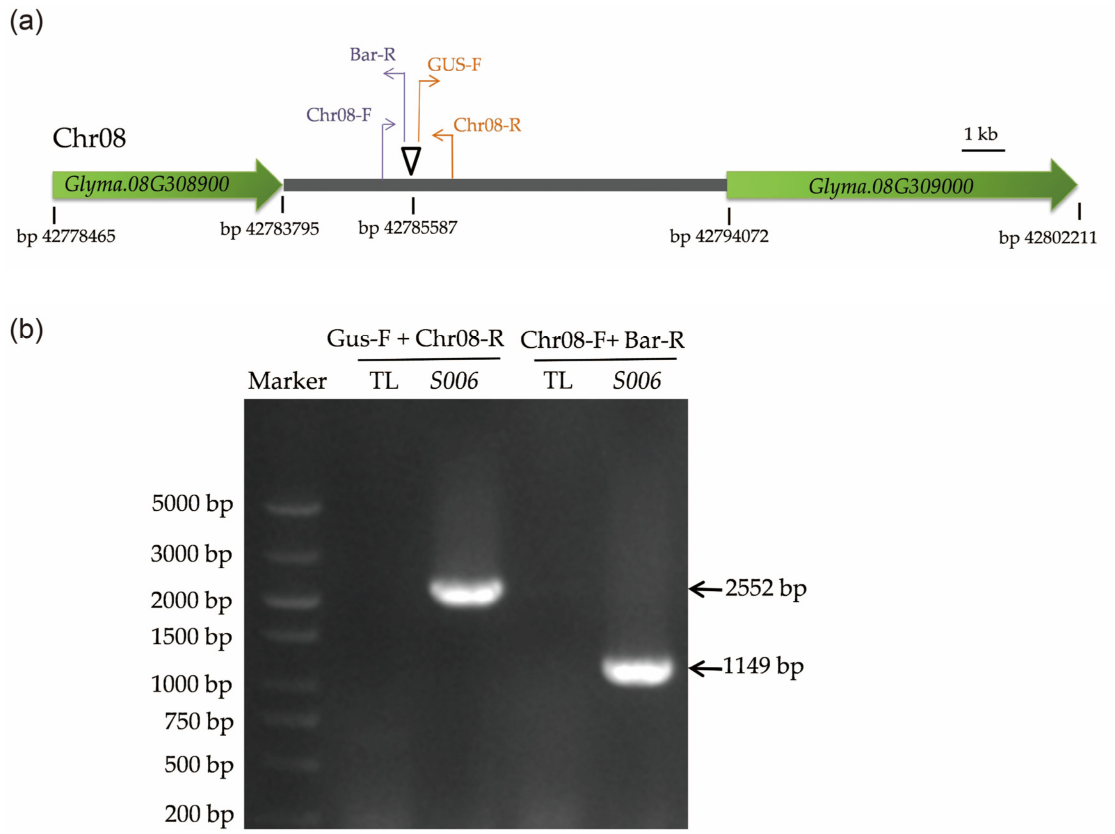

Figure S5. The schematic diagram and identification of the T-DNA insertion in the *S006* mutant. (a) The schematic diagram for the T-DNA insertion in the *S006* mutant; (b) gel determination for the T-DNA insertion in the *S006* mutant.
