# Supplementary figures and images for: Novel Seed Size: A Novel Seed-Developing Gene in Glycine max
Source: Int J Mol Sci. 2023 Feb 20;24(4):4189. doi: 10.3390/ijms24044189 (PMC9967547; doi:10.3390/ijms24044189)

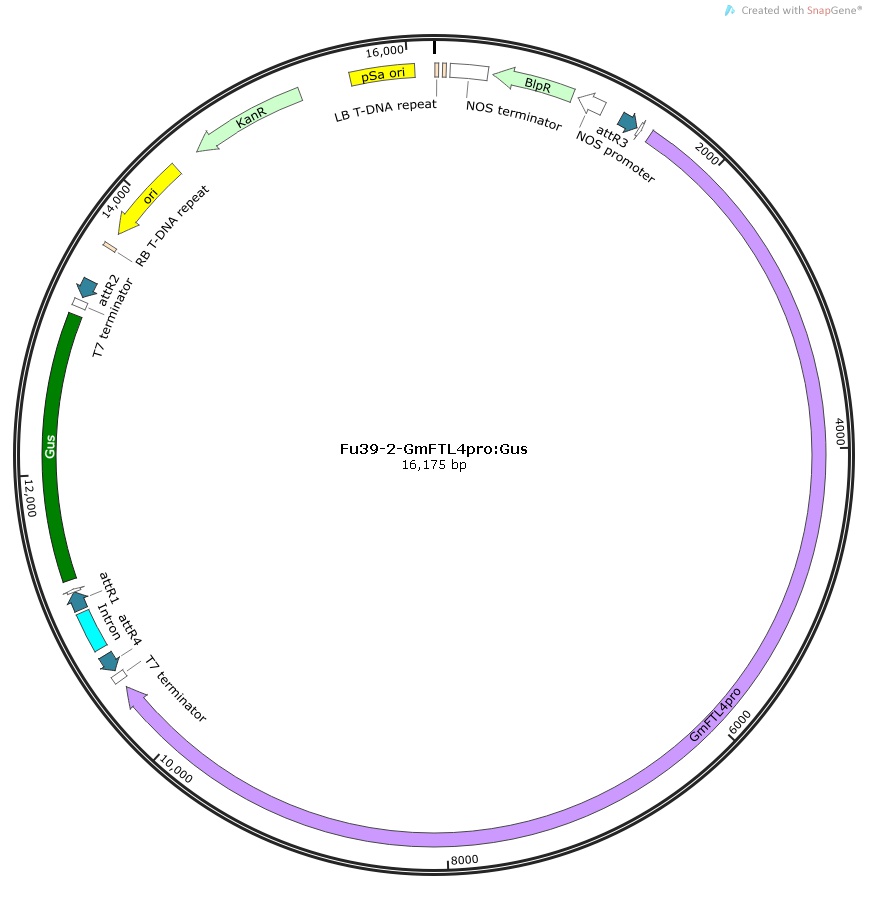

Supplement: Supplementary file 1 [file ijms-24-04189-s001.zip › Figure S1.jpg]

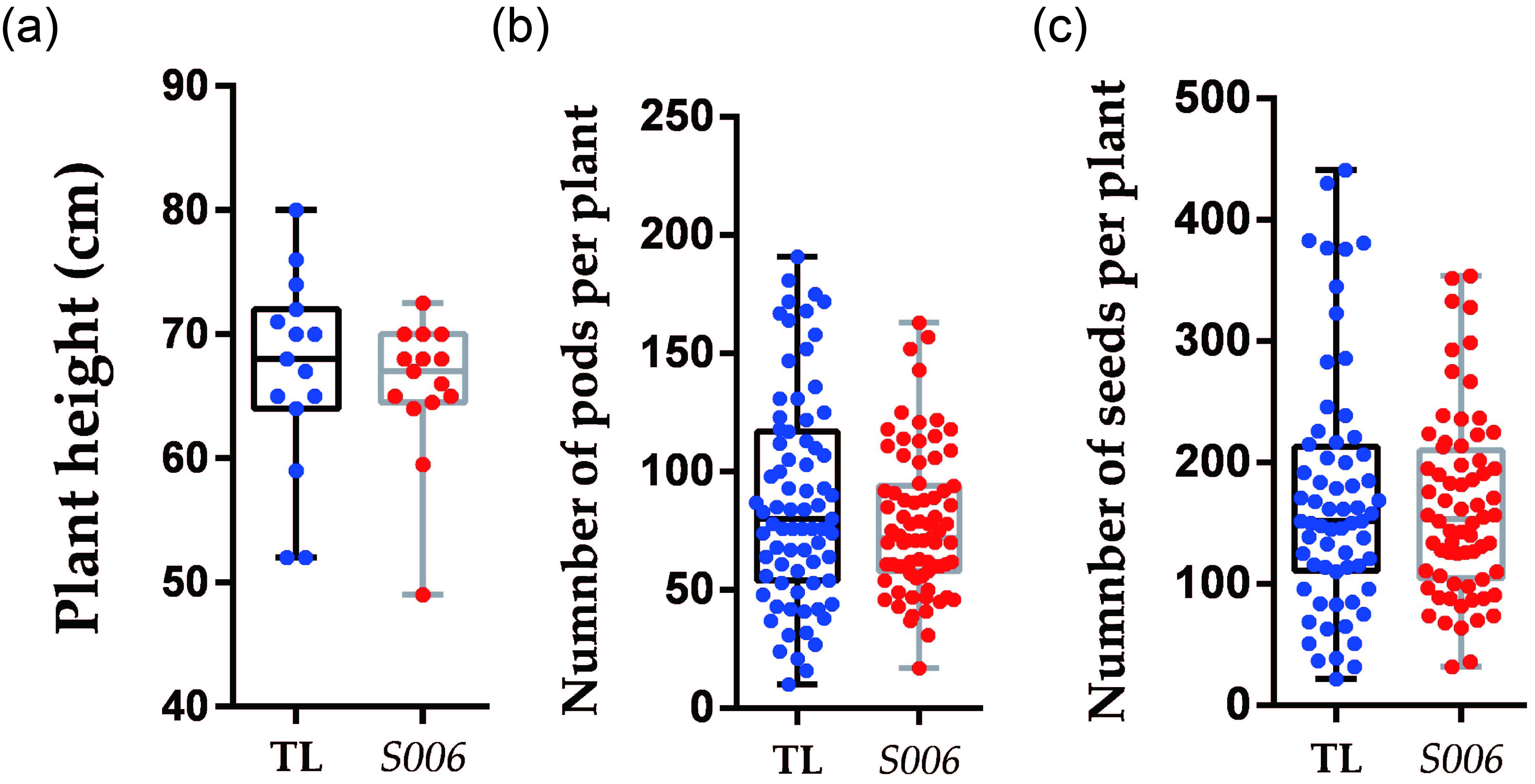

Supplement: Supplementary file 1 [file ijms-24-04189-s001.zip › Figure S2.jpg]

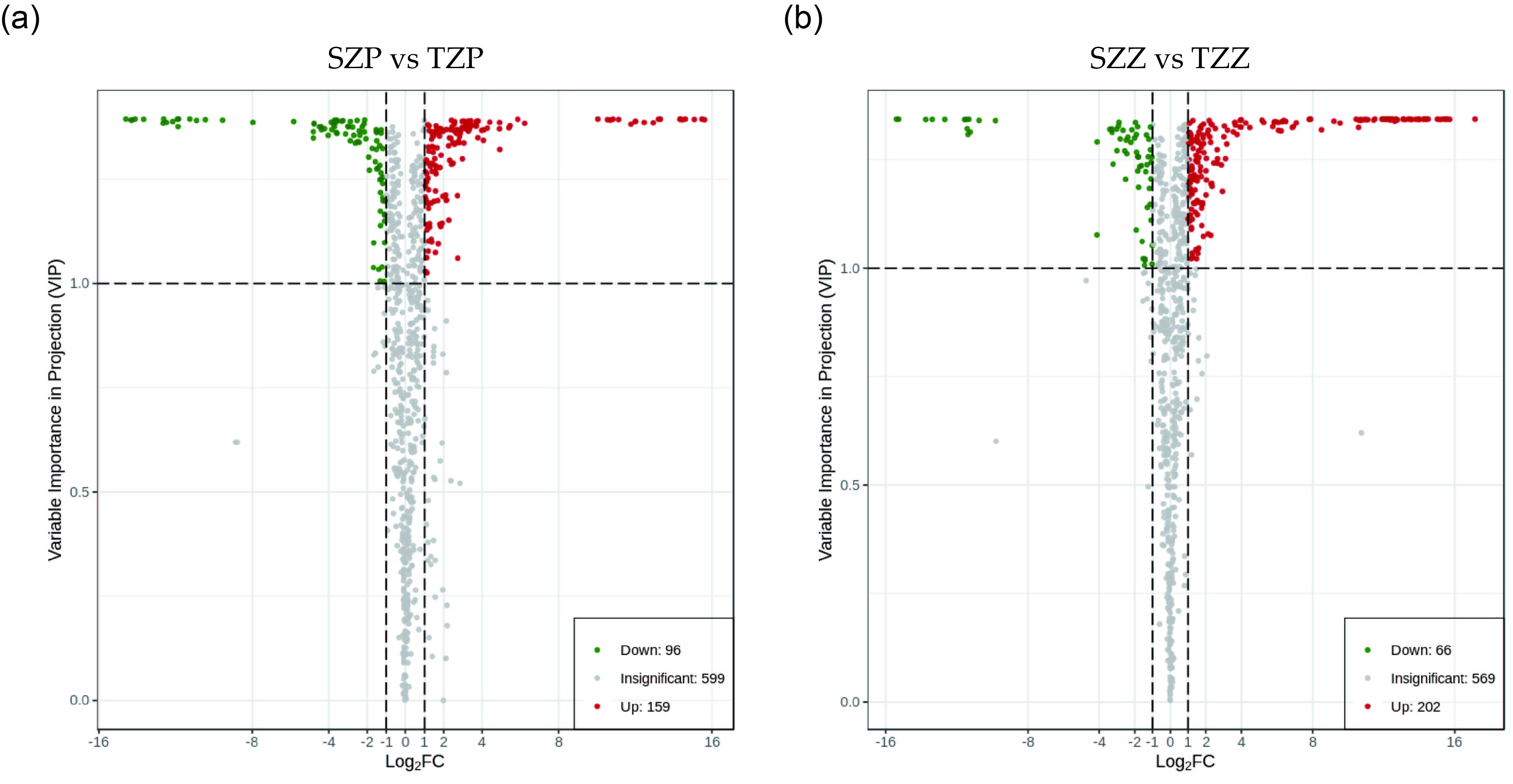

Supplement: Supplementary file 1 [file ijms-24-04189-s001.zip › Figure S3.jpg]

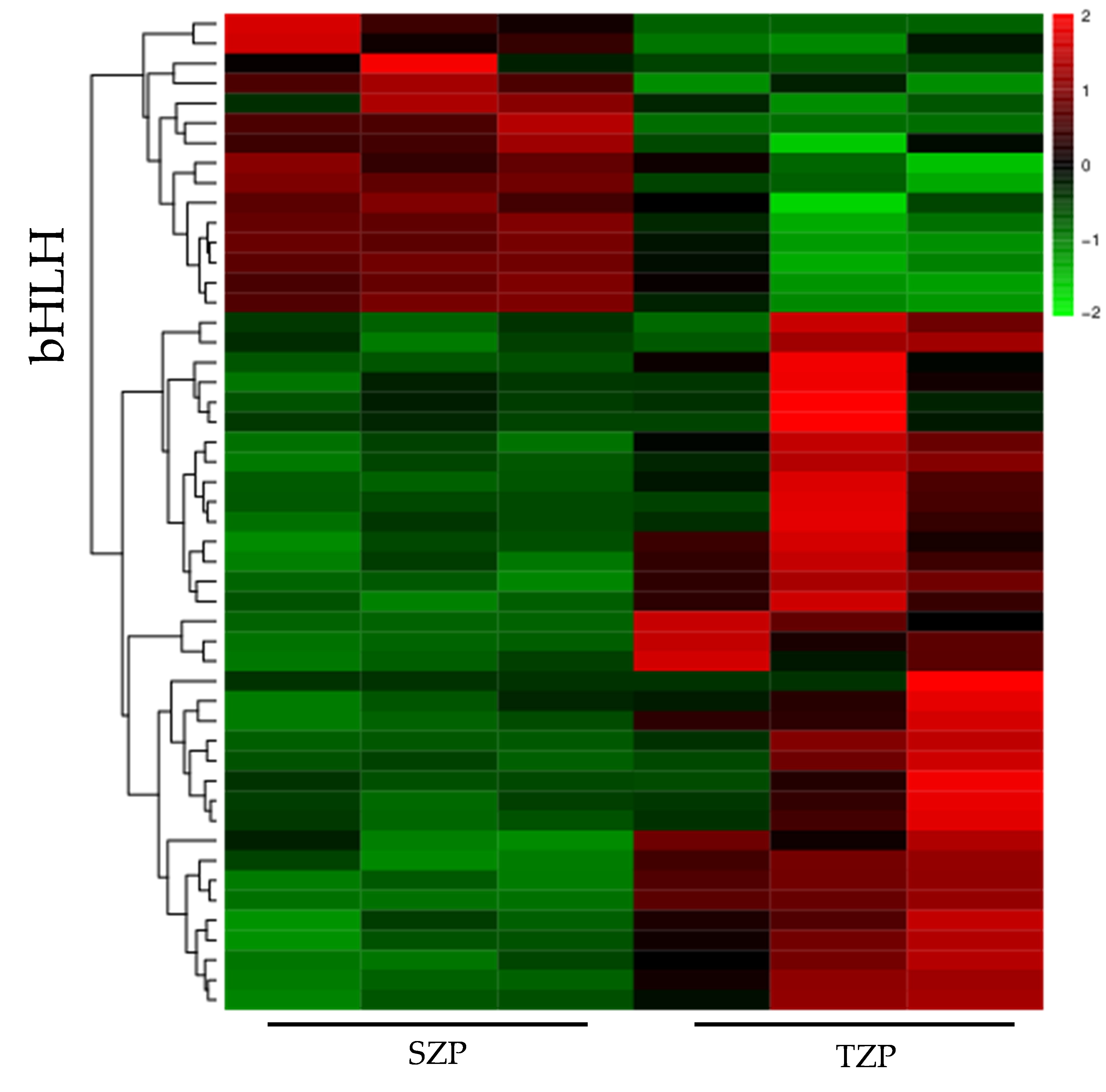

Supplement: Supplementary file 1 [file ijms-24-04189-s001.zip › Figure S4.jpg]

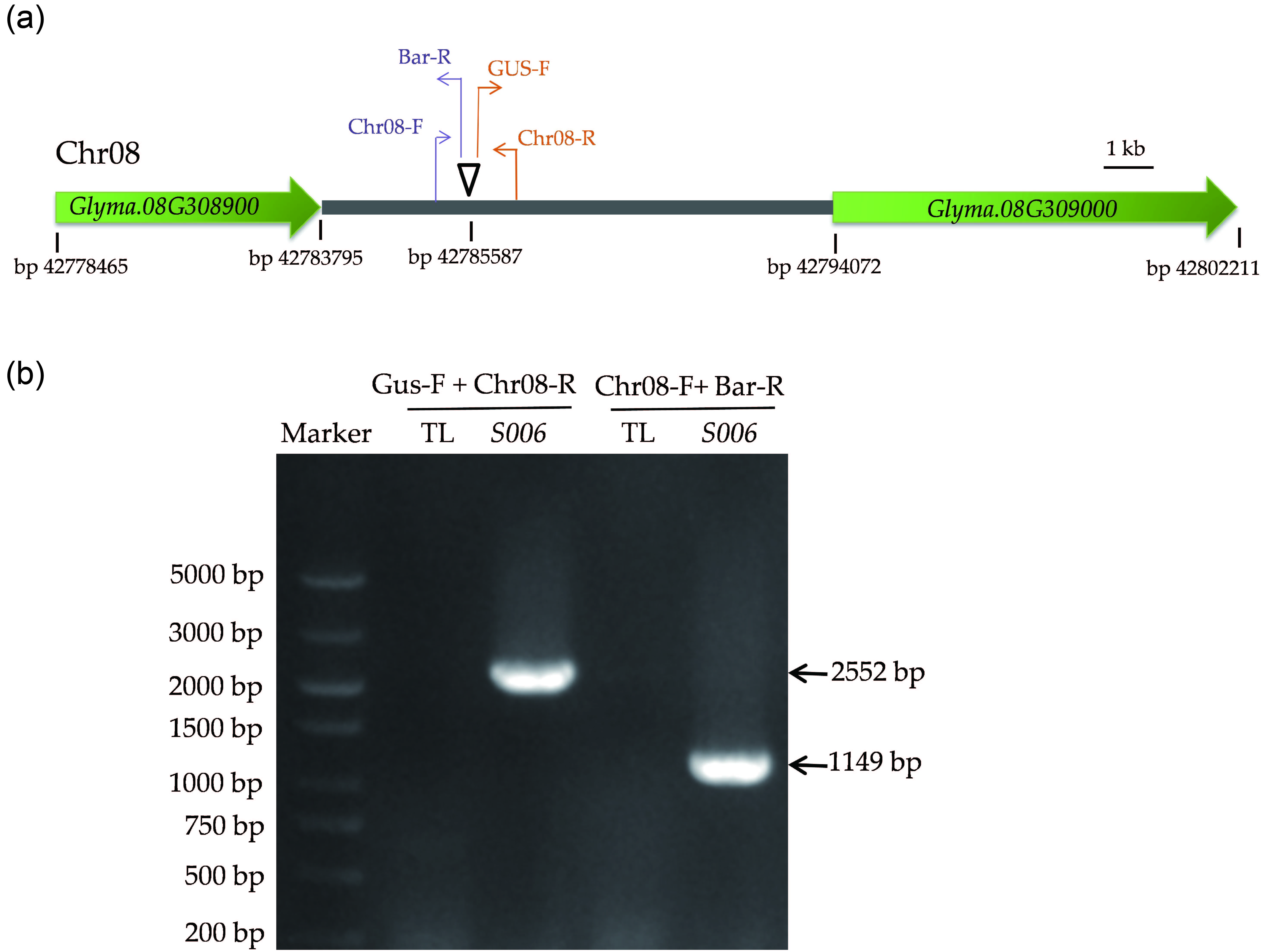

Supplement: Supplementary file 1 [file ijms-24-04189-s001.zip › Figure S5.jpg]
